# Boron nitride nanotubes as containers for targeted drug delivery of doxorubicin

**DOI:** 10.1007/s00894-020-4305-z

**Published:** 2020-02-08

**Authors:** Marjan A. Nejad, Philipp Umstätter, Herbert M. Urbassek

**Affiliations:** Fachbereich Physik und Forschungszentrum OPTIMAS, Universität Kaiserslautern, Erwin-Schrödinger-Straße, D-67663 Kaiserslautern, Germany

**Keywords:** Molecular dynamics, Nanotubes, Doxorubicin, Targeted drug delivery systems

## Abstract

Using molecular dynamics simulations, the adsorption and diffusion of doxorubicin drug molecules in boron nitride nanotubes are investigated. The interaction between doxorubicin and the nanotube is governed by van der Waals attraction. We find strong adsorption of doxorubicin to the wall for narrow nanotubes (radius of 9 Å). For larger radii (12 and 15 Å), the adsorption energy decreases, while the diffusion coefficient of doxorubicin increases. It does, however, not reach the values of pure water, as adsorption events still hinder the doxorubicin mobility. It is concluded that nanotubes wider than around 4 nm diameter can serve as efficient drug containers for targeted drug delivery of doxorubicin in cancer chemotherapy.

## Introduction

Among available anticancer drugs, doxorubicin—with the trade name Adriamycin—plays an important role. It has been used to treat a wide array of cancers—such as breast, liver, and osteosarcoma cancers—for more than 40 years [1], despite several severe side effects like cardiotoxicity which is the most dangerous one [2]. Doxorubicin acts on the DNA by binding to AT and GC sequences; as a consequence, cancer cells are stopped growing [3].

The method of “targeted drug delivery” was established to deliver drugs directly to the cancer cells that are the targets of the treatment [4]. They make use of appropriate carrier systems (nanocontainers) that must both be biocompatible and release the drug in a controlled way [5].

Boron nitride nanotubes are similar to carbon nanotubes, but carbon atoms are substituted by boron and nitrogen atoms. They can be produced by rolling a boron nitride nano-sheet and have the same structure as carbon nanotubes [6]. This nanomaterial has a great potential for use in biomedicine because of its biocompatibility, chemical inertness, high thermal stability, and unique electrical and optical characteristics [7, 8]. Ferreira et al. [9] have shown in an experimental study that folic acid–functionalized boron nitride nanotubes are good candidates for targeted cancer therapy because of their high cellular uptake.

Among the special medical applications of boron, the boron neutron-capture therapy (BNCT) stands out as a radiotherapy that has been used since the 1950s to treat cancers. The mechanism of this therapy is based on boron neutron-capture nuclear reactions [10]. In this therapy, boron is injected into the body and moves with the blood circulation; when it reaches the tumor site, the tumor will be irradiated by thermal neutrons. The nuclear reaction with boron produces *α* particles that have a short path length and a high energy deposition in tissue; they destroy the cancer cells while sparing the normal cells [11]. BNCT could be a promising therapy for localized tumors. Despite good results, BNCT is still not a common cancer therapy because of some difficulties like finding biocompatible boron compounds and thermal neutron sources. L-para-boronophenylalanine (BPA), C$_{9} H_{12} {~}^{10} \mathit {BNO}_{4}$, and sodium mercaptoundecahydro-closo-dodecaborate (BSH), Na$_{2} {~}^{10}\textit {B}_{12}\textit {H}_{11}$SH, were used early as boron compounds in the treatment trial of high-grade gliomas in the 1960s [12]. Geninatti-Crich et al. [12] demonstrated in their study that boronated compounds can be used for image-guided boron neutron-capture therapy. Recently, the Kyoto University research center used BNCT to treat patients with vulvar melanoma and genital extramammary Paget’s disease. They reported that the treatment was successful and they recommend this therapy for this kind of cancers [13]. In another study, Ferreira et al. [14] showed that boron nitride nanotubes might be good candidates for BNCT therapy.

Nanotubes are suitable candidates for smart drug delivery because they allow controlled drug release in the appropriate sites. In an early study [15], Ali-Boucetta et al. showed that copolymer-coated multiwalled carbon nanotubes can serve as good candidates for doxorubicin drug delivery. More recently, Emanet et al. [16] demonstrated that boron nitride nanotubes can enhance the biocompatibility and mechanical strength of the composites used for tissue engineering.

For a proper understanding, the diffusion of the drug in the nanotube as well as its adhesion to the walls must be studied. Atomistic simulations based on molecular dynamics have proven useful to guide our understanding, since available force fields allow to describe the interatomic interactions governing the drug behavior quite well, and thus both the binding of the drug to the container wall as well as its diffusion along the wall and out of the container can be assessed. Thus, for the example of carbon nanotubes, molecular dynamics studies described the encapsulation and release of cisplatin from carbon nanotubes [17, 18]. In a first-principles study [19], the potential of boron nitride nanotubes as platinum drug molecule carriers was demonstrated. Another classical molecular dynamics study presented results on the encapsulation and release of cisplatin drug molecules from boron nitride nanotubes [20].

The performance of nanotubes as containers of doxorubicin does not appear to have been studied by simulation up to now. However, the stability and diffusion of doxorubicin along graphene sheets and graphene oxide functionalized with polyethylene glycol were studied by Wang et al. [21]. Shan et al. [22] focused on the interaction of doxorubicin with chitosan oligosaccharides to shed light on the encapsulation of doxorubicin by long-chained chitosan oligosaccharides, and Zhang et al. [23] studied the interaction of doxorubicin with P-glycoprotein, a transmembrane protein, to identify binding sites and transport of doxorubicin inside the protein.

In the present study, we use classical molecular dynamic modeling methods to investigate the diffusion of doxorubicin in boron nitride nanotubes of varying radius. In addition, we determine the interaction energy between doxorubicin and the nanotube wall in order to characterize the influence of wall adsorption during the diffusion process. Thus, our results allow to shed light on the potential use of boron nitride nanotubes as drug containers for doxorubicin.

## Methods

We constructed boron nitride nanotubes with the help of the nanotube-builder plug-in of VMD [24]. Three nanotubes with radii *R* = 9.04, 12.43, and 15.20 Å were built; all of them have a length of 40 Å and their edges have an armchair configuration. In the notation of chiral indices [25, 26], they are characterized as (13, 13), (18, 18), and (22, 22).

The atomic positions of B and N in the nanotubes were considered to be fixed throughout the simulation. The interaction of B and N with water was taken from the study of Wu et al. [27]. They determine the van der Waals (vdW) attractions of B (N) to 0.098 (0.121) kcal/mol and the charges to + 0.3|*e*| (− 0.3|*e*|).

**Fig. 1:**
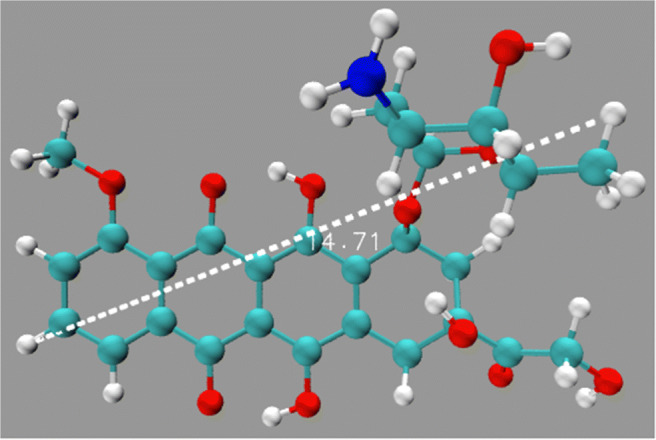
Doxorubicin molecule. White: hydrogen, red: oxygen, blue: nitrogen, cyan: carbon. The molecule size is indicated

The conformation of doxorubicin, C_27_*H*_29_*NO*_11_ (68 atoms), was obtained from the PDB database [28]. Figure 1 gives an atomistic representation of this drug molecule; its longest extension is about 1.5 nm. Doxorubicin has a planar part, which is based on a linear sequence of four aromatic rings, similar to tetracene; additional side chains, among them another aromatic ring, give it a three-dimensional structure. Interatomic interactions are modeled with the CHARMM General Force Field (CGENFF) [29]; the CHARMM-GUI [30] web-based graphical user interface was used to make the parameters of doxorubicin available within this force field. Doxorubicin is flexible within this force field. The Lennard-Jones parameters describing the vdW interaction of doxorubicin with B and N were obtained from the usual Lorentz-Berthelot mixing rules. The cut-off for both the electrostatic and the vdW interaction was set to 12 Å. Note that no covalent bonds are formed between doxorubicin and the boron nitride nanotube; that is, we assume that since all bonds in the boron nitride nanotube are saturated, interaction with doxorubicin can be modeled via the vdW interaction. This may be different at the tube edges; however, we made sure that the nanotube was sufficiently long that doxorubicin never approached the edges in our simulations.

Initially, we put the drug molecule in the middle of the nanotube and the entire system is solvated in TIP3P [31, 32] water. Periodic boundary conditions are employed to get rid of any boundary effects. The system is subjected to an energy minimization procedure for 3 × 10^4^ time steps and is then equilibrated for 1 ns at a temperature of 300 K and constant pressure of 1 bar, such that the water-nanotube interface and also the doxorubicin hydration shell could establish. During the equilibration process, the center of mass of the drug molecule was constrained.

After the system was prepared, diffusion simulations of 3 ns duration in an NPT ensemble were started; for each nanotube, we perform 20 simulations, which vary among each other by the thermal fluctuations during the run. The timestep amounted to 2 fs. We use the software NAMD 2.10 [33] for performing the molecular dynamics simulations. VMD 1.9.3 [34] and Tachyon [35] were used to render the adsorption snapshots.

## Results and discussion

### Diffusivity of doxorubicin in bulk water

Since the encapsulated drugs should release in the body blood circulation, we need to know the diffusivity of the drug molecule in the nanotube and also in the pure fluid environment. As a reference case, we first determine the diffusion coefficient *D* of doxorubicin in bulk water. This is done with the help of the Einstein relation [36]

1 $$ \langle { r}^2(t) \rangle = 6D t , $$

which describes the mean square displacement (MSD) 〈*r*^2^(*t*)〉 of a drug molecule as a function of time *t*.

In detail, we proceed as follows [37–39]. Our diffusion runs provide data for the individual particle trajectories *r*_*i*_(*t*), where *r* denotes the center of mass of the doxorubicin molecule, *i* counts the simulations performed, $i=1, \dots , N_{\text {tra}}$, and *N*_tra_ = 20 simulations. For each simulation *i*, we choose *N*_sta_ = 1500 time windows of length 100 ps, starting at a randomly chosen initial time *t*_*ij*_, where $j=1, \dots , N_{\text {sta}}$. This double averaging over individual trajectories and time windows allows to calculate the MSD as

2 $$ \langle { r}^2(t) \rangle = \frac{1}{N_{\text{tra}}} \sum\limits_{j=1}^{N_{\text{tra}}} \frac{1}{N_{\text{sta}}} \sum\limits_{i=1}^{N_{\text{sta}}} \left| { r}(t_{ij}+t)-{ r}(t_{ij})\right|^2 . $$

To the data, we then fit a function *f*(*t*) = *a* + *bt* and the diffusion coefficient can be calculated as *D* = *b*/6.

**Fig. 2:**
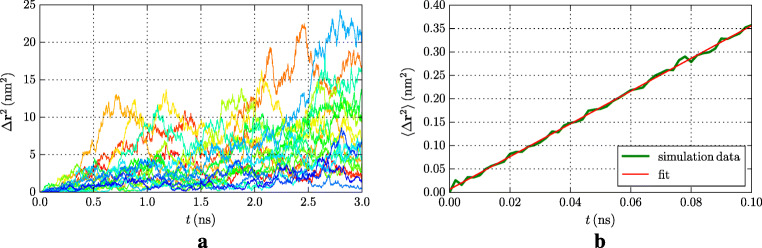
Diffusion of doxorubicin in bulk water. **a** MSD of 20 individual 3-ns diffusion runs. **b** Average over the individual runs, compared to a linear fit line

Figure 2a shows the time dependence of the individual trajectories, while Fig. 2b displays the average value. After averaging, the data allow a fit to Eq. (1) resulting in a diffusion coefficient of *D* = 583.1 ± 3.9 *μ*m^2^/s.

We are not aware of any experimental or previous theoretical determination of the diffusion coefficient of doxorubicin in water.

For comparison, we note that the self-diffusion coefficient of water in the TIP3P potential amounts to 5100 *μ*m^2^/s at 300 K [40]. From Stokes’ law, we expect a dependence of the diffusion coefficient proportional to 1/*r*, where *r* is the molecule “radius.” Since the vdW diameter of water is *r* = 2.82 Å [41], while for doxorubicin it amounts to *r* = 15 Å, we expect a reduction of the diffusion coefficient for doxorubicin by a factor of around 5, in rough agreement with our results. The remaining quantitative discrepancy is owed to the inadequacy of the macroscopic Stoke’s law in the microworld as well as to the non-spheric structure of both the water and the doxorubicin molecule.

We note that we chose the length of the nanotube considered in this work (40 Å) from these diffusion studies. Since the diffusivity in the nanotube is expected to be smaller than that in pure water, during the time of 3 ns, no escape of doxorubicin from a nanotube of length 40 Å is expected, and indeed none was observed.

### Diffusivity of doxorubicin in the boron nitride nanotubes

The diffusivity of doxorubicin in the boron nitride nanotubes was calculated in a similar way as in bulk water; however, since here we are only interested in the motion of the molecule along the nanotube axis, we use the one-dimensional version of Einstein’s relation

3 $$ \langle z^2(t) \rangle = 2D t , $$

where *z* denotes the drug molecule coordinate along the axis of the cylindrical nanotube. Since we did not see any escape of drug from nanotube during all simulation times, we believe that this approach is quite well defined for calculating the diffusion coefficients alongside the nanotube’s axis.

**Fig. 3:**
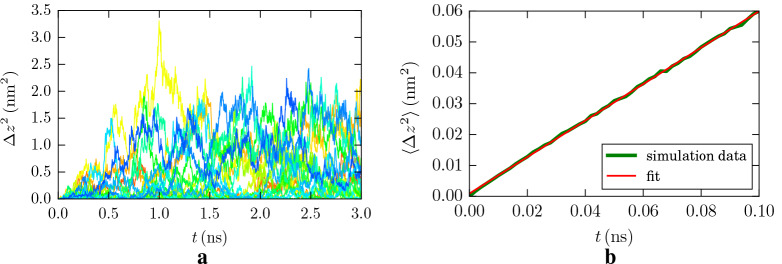
Diffusion of doxorubicin in a boron nitride nanotube of radius 12.4 Å. **a** MSD of 20 individual 3-ns diffusion runs. **b** Average over the individual runs, compared to a linear fit line

We display in Fig. 3a the trajectories of individual doxorubicin molecules in the boron nitride nanotube of middle size, radius 12.4 Å, as an example. The size of the squared displacements is now considerably smaller than in bulk water, indicating that the boron nitride nanotube hinders the motion of doxorubicin. In some of the trajectories, the doxorubicin molecule appears to remain stuck at certain positions for longer periods of time; these are adsorption events. However, also swift free motion from one position to another is observed, where the molecule is only weakly bound to the nanotube. In no simulation, we saw doxorubicin escaping the nanotube.

An averaging over the trajectories, as described above, Eq. (2), is plotted in Fig. 3b. The mobility is well described by a diffusive law as in Eq. (3) with a diffusion coefficient of *D* = 295.6 ± 0.7 *μ*m^2^/s. A similar diffusive behavior is found for the other nanotubes, with diffusion coefficients of *D* = 245.1 ± 1.6 (357.1 ± 1.6) *μ*m^2^/s for a nanotube radius of 9.0 (15.2) Å.

**Fig. 4:**
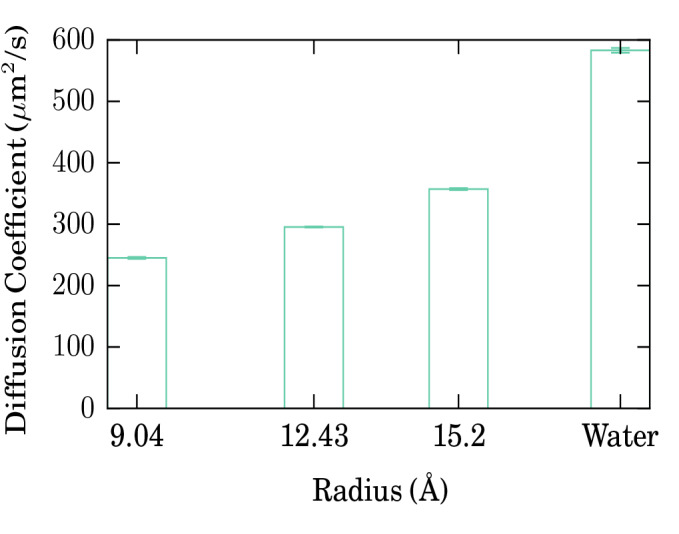
Diffusion coefficients of doxorubicin in different nanotubes and in water. The error bars as obtained from the uncertainty of the fit of the slope *D* in Eq. 3 are smaller than the linewidth

The data are assembled in Fig. 4 and compared to the diffusivity of doxorubicin in bulk water. The increase of the diffusion coefficient with nanotube radius is evident. Still, even for the largest radius, the diffusivity in the nanotube is considerably below the value for pure water. Such a dependence of the diffusion coefficient on the radius of the container was also observed in previous studies, such as the diffusion of cisplatin in silica nanopores [42, 43]. This dependence has been attributed to the temporary adsorption of the drug molecule to the container wall. This phenomenon will be investigated in the following section.

### Adsorption at the nanotube walls

**Fig. 5:**
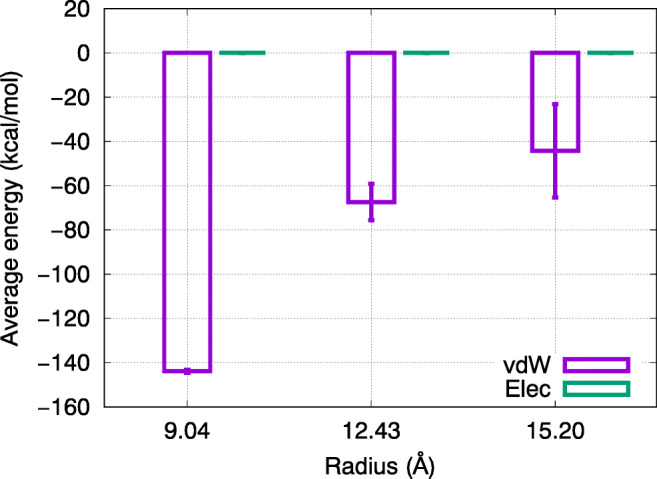
Average electrostatic (“Elec”) and vdW (“vdW”) energies between the doxorubicin molecule and the nanotube wall during the 3-ns diffusion process as a function of the nanotube radius. Error bars are obtained from the fluctuations of the energies along the trajectories

We calculate the interaction energy between doxorubicin and the nanotube wall. The vdW and electrostatic contributions are evaluated separately. Figure 5 shows these data averaged over the 20 simulations and over the entire trajectories.

The averaged electrostatic energy values are tiny, of the order of 10^− 3^ kcal/mol, and thus 4 orders of magnitude below the vdW energies. This is astonishing, since both the boron nitride nanotube and the drug molecule carry partial charges, even if they are net neutral. However, the charges in the boron nitride nanotube tend to screen each other, since in the hexagonal arrangement of B and N, always unlike charges are close to each other and no large charged patches show up. Hence, at some distance—of the order of a few atomic distances—the charge distribution appears smeared out and the electrostatic interaction becomes negligible. In addition, momentary electrostatic interactions may be larger in magnitude and reach values of up to ± 0.3 kcal/mol; however, since interactions may have either sign, they cancel on time-averaging.

The vdW interaction, however, is always attractive. Its averaged magnitude strongly decreases with nanotube radius, indicating that the strength of adsorption decreases with increasing nanotube radius. This is in line with the increase of the diffusion coefficient with nanotube radius *R* discussed above. Also, the fluctuations along the trajectory and between different simulation runs increase with increasing nanotube diameter. This is caused by the fact that in narrow nanotubes, the doxorubicin cannot easily change its adsorption state due to the wall constraints, while in wide nanotubes, doxorubicin can adsorb and desorb repeatedly and also change its orientation towards the container wall and hence its adsorption energy.

We will now discuss selected individual trajectories in order to shed more light on the adsorption state of the doxorubicin and its variation with time. Here, we will omit the discussion of the electrostatic interaction, since—as discussed above—it is tiny and does not influence the adsorption or diffusion behavior.

**Fig. 6:**
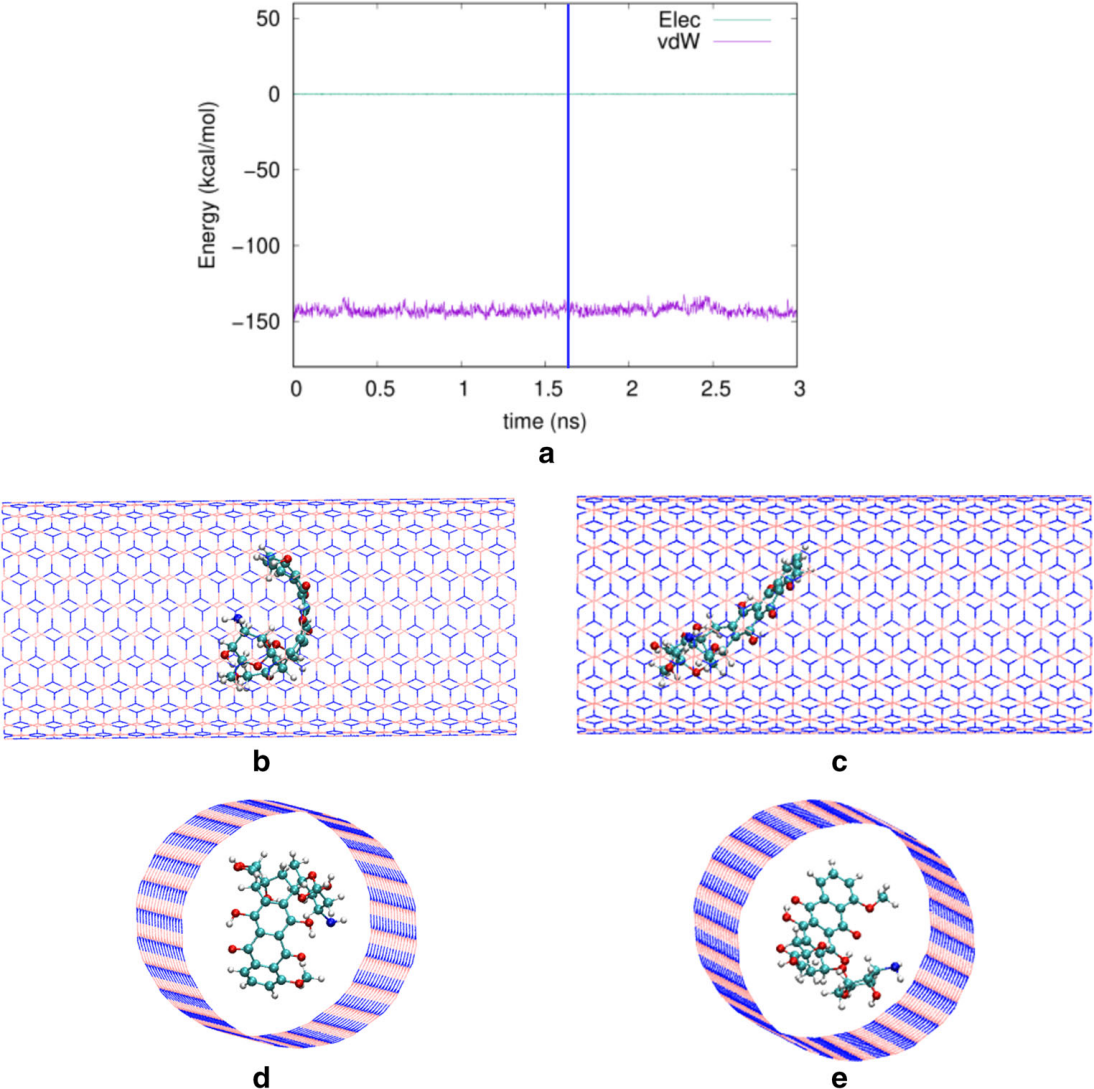
**a** Time evolution of the electrostatic (“Elec”) and vdW energies between the doxorubicin molecule and the boron nitride nanotube of radius 9.0 Å. Side views and perspective views of the system are provided at the beginning, *t* = 0 ps (**b**, **d**) and at 1680 ps (**c**, **e**). This time is marked in **a** by a vertical blue line

Figure 6a shows the time evolution of the adsorption energy for the narrowest nanotube. Here, the vdW energy stays almost constant; its value agrees with the ensemble average shown in Fig. 5, − 143.9 kcal/mol. Figure 6b–e indicate the position and orientation of the doxorubicin initially and at a later time during the simulation. In order to allow for a better three-dimensional visualization, the positions are shown both in a side-on and a perspective view. We see that the orientation of doxorubicin has changed somewhat during the simulation run. While initially the planar part of doxorubicin was oriented perpendicular to the nanotube axis, it has somewhat rotated during the simulation. Also, the center of mass has clearly moved to the left-hand side (see Fig. 6c). Interestingly, also considerable deformation of doxorubicin is observed in that the initially planar orientation of the aromatic rings becomes curved (see in particular Fig. 6b and d). Note that despite these changes the value of the vdW energy is virtually unchanged. This is because the large interaction range of the vdW force sums over all atoms nearby the nanotube wall; in the narrow nanotube geometry, all possible doxorubicin orientations give similar values of the vdW interaction. In the narrowest nanotube, we observed from the videos in all trajectories that the nanotube tries to align the planar tetracene component of the doxorubicin structure towards the nanotube wall.

**Fig. 7:**
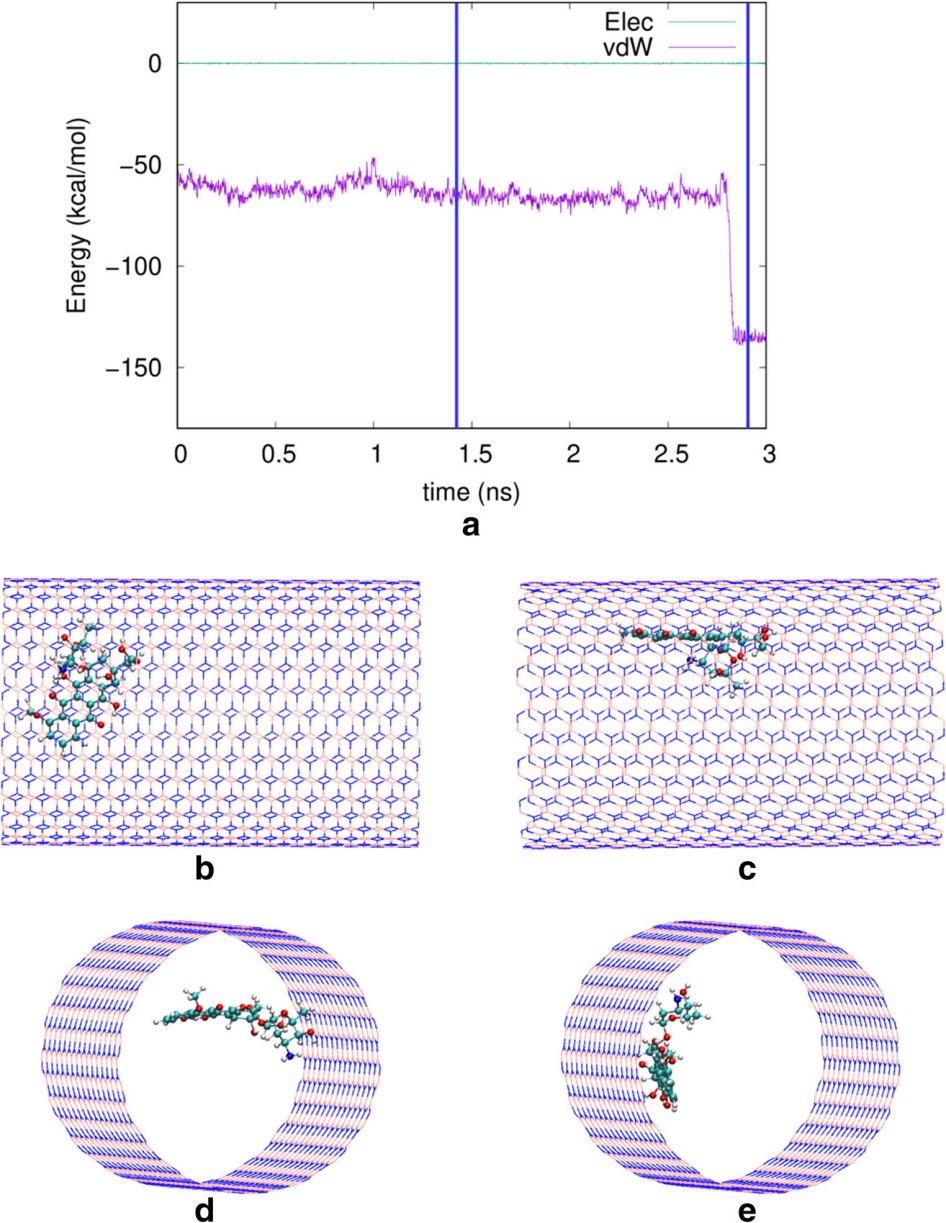
**a** Time evolution of the electrostatic (“Elec”) and vdW energies between the doxorubicin molecule and the boron nitride nanotube of radius 12.4 Å. Side views and perspective views of the system are provided at 1446 ps (**b**, **d**) and at 2930 ps (**c**, **e**). These times are marked in **a** by vertical blue lines

For a larger radius, 12.43 Å, the time evolution is exemplarily shown in Fig. 7. Figure 7a shows that over a long time, in this trajectory, the doxorubicin moves while approximately retaining the average energy of Fig. 5, − 67.4 kcal/mol. Figure 5b shows that it can diffuse quite long distances—up to close to the end of the nanotube—in this adsorbed state. The doxorubicin orientation is similar to what was seen in the narrow nanotube (Fig. 6). However, since now only part of the molecule is close to the wall, the adsorption energy is considerably smaller than that in the narrow nanotube. Then, at around 2.7 ns, doxorubicin changes its orientation and comes flat down to the wall, increasing the adsorption energy to around 137 kcal/mol. This value is now closer to the adsorption energy in the narrow nanotube, since now a considerable fraction of doxorubicin is within the range of the nanotube vdW interaction.

**Fig. 8:**
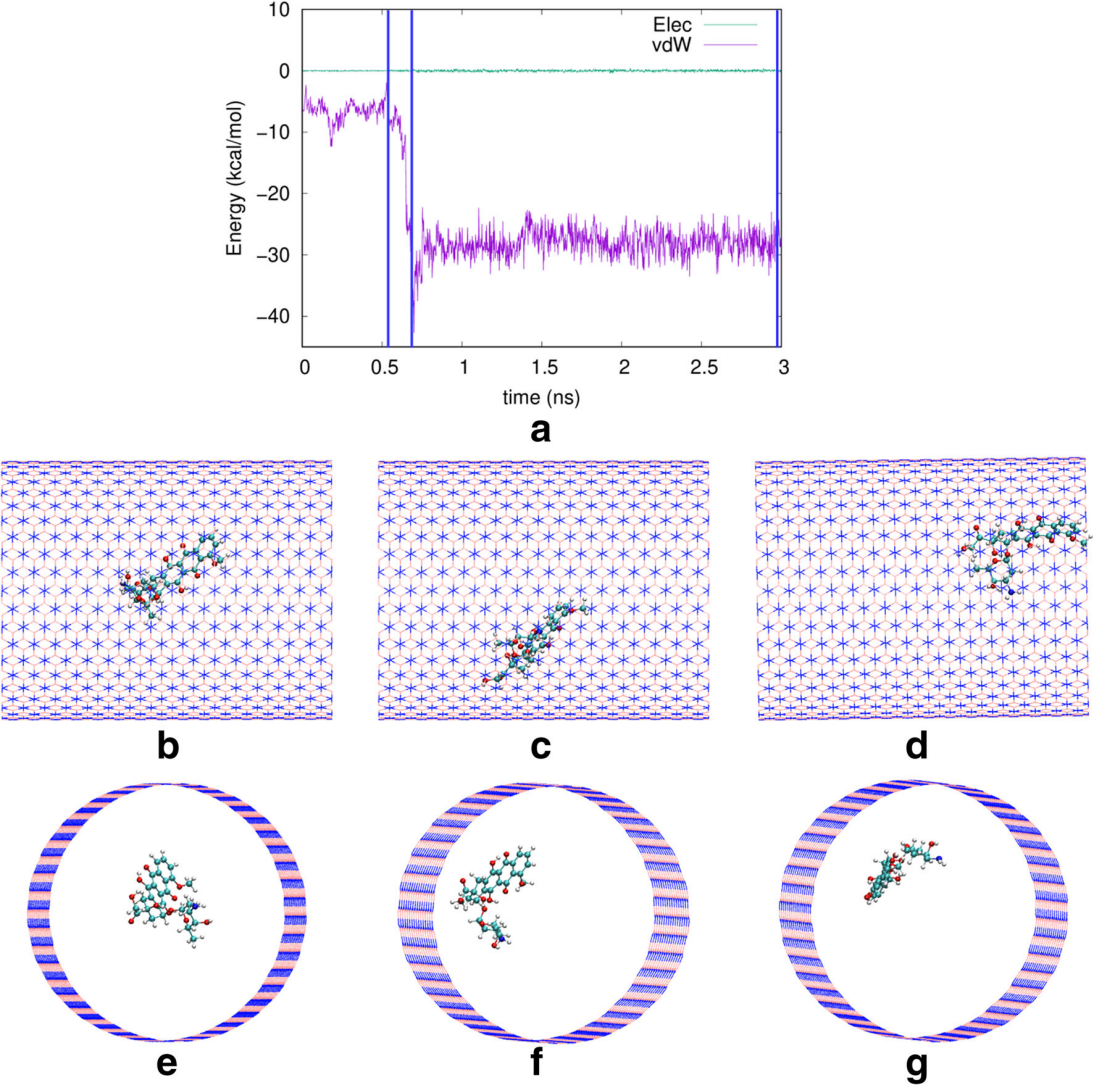
**a** Time evolution of the electrostatic (“Elec”) and vdW energies between the doxorubicin molecule and the boron nitride nanotube of radius 15.2 Å. Side views and perspective views of the system are provided at at 528 ps (**b**, **e**), at 700 ps (**c**, **f**), and at 2965 ps (**d**, **g**). These times are marked in **a** by vertical blue lines

Finally, the snapshots shown in Fig. 8 give a representative example of the motion of doxorubicin in the widest nanotube studied, *R* = 15.2 Å. In its initial position in the middle of the nanotube, the adsorption energy is small in magnitude, amounting to only − 1.96 kcal/mol (Fig. 8b and e). As the molecule approaches the wall, it becomes more strongly bound. In the two conformations shown at the times of 700 and 2965 ps, the vdW adsorption energy reaches values of − 42.70 kcal/mol and − 24.52 kcal/mol, respectively. In this weakly bound state, the mobility of doxorubicin is high and it can even approach the nanotube edge (Fig. 8g). However, we never saw it escape from the nanotube; presumably, its attractive energy to the nanotube edges was still too high to allow it to leave the nanotube.

We note that in another trajectory (not shown), we found doxorubicin to stay in close contact to the wall, assuming a conformation with a vdW adsorption energy of − 132.48 kcal/mol; in this state, of course, its motion was more strongly hindered. The possible occurrence of more deeply bound trajectories is evident from the large standard deviation of the adsorption energy shown in Fig. 5. However, the probability of such trajectories is small.

We conclude that the range of nanotube radii studied here includes the case where doxorubicin is closely constrained (*R* = 9.0 Å) up to the case where it is quite free to move (*R* = 15.2 Å). With increasing radius, the average vdW adsorption energy decreases; however, individual trajectories experience doxorubicin positions where strong bonding to the wall occurs, but also desorption events take place after which doxorubicin is free to diffuse in the middle of the nanotube. We therefore expect that for even larger radii than simulated by us, the doxorubicin diffusion coefficient will become even larger and approach the value of bulk water. On the other side, we expect that doxorubicin will hardly fit in smaller nanotubes than those simulated here; and if so, then its mobility will be close to zero.

In none of our simulations, we observed escape of the doxorubicin from the nanotube; evidently, the vdW attraction is too strong even in the widest nanotube considered here. We expect that the nanotube radius must be larger than the values studied in the present work in order to allow for doxorubicin escape.

## Conclusions

From our simulations of doxorubicin diffusion in boron nitride nanotubes, we can draw the following conclusions.

1. Since doxorubicin is quite large (1.5 nm in its longest extension), the nanotube diameter must be beyond this size to allow mobility.
2. With increasing nanotube radius, diffusivity strongly increases. Concomitantly, the adsorption energy decreases. From an extrapolation of our data, it may be expected that only for nanotube diameters beyond around 2.8 nm (based on a linear extrapolation of the data provided in Fig. 4), the diffusion coefficient will approach values close to that in pure water.
3. For the smallest nanotube diameter considered here, the doxorubicin molecule always remains bound to the wall. Here, vdW attraction strongly dominates, while electrostatic forces are negligible. The reason for this is that doxorubicin is net neutral, and also the nanotube surface appears uncharged at some distance where the regular charge distribution provided by the B and N atoms has been smeared out.
4. With increasing nanotube radius, the motion of doxorubicin can hence be characterized as a sequence of adsorption and desorption phases—similar to what we found for small molecules in silica nanotubes [42–44]—while for small nanotube radii, it can rather be described as a motion along the surface, since the binding to the surface is never destroyed.

Our study thus corroborates the view that boron nitride nanotubes may be useful as containers for drug molecules, such as the doxorubicin molecule considered here. Previous studies already pointed out the benefits of the biocompatibility and mechanical strength of boron nitride nanotube containers [7–9]. Our findings add that nanotube diameters larger than around 4 nm are needed to allow for efficient diffusion of the drug within the nanotube, and to reduce the adsorption of the drug molecules from the container walls.
