# Correction to: Boron nitride nanotubes as containers for targeted drug delivery of doxorubicin

**DOI:** 10.1007/s00894-021-04832-y

**Published:** 2021-07-07

**Authors:** Marjan A. Nejad, Philipp Umstätter, Herbert M. Urbassek

**Affiliations:** Fachbereich Physik und Forschungszentrum OPTIMAS, Universität Kaiserslautern, Erwin-Schrödinger-Straße, D-67663 Kaiserslautern, Germany

**Correction to: Journal of Molecular Modeling (2020) 26: 54**

10.1007/s00894-020-4305-z

The article “Boron nitride nanotubes as containers for targeted drug delivery of doxorubicin”, written by Nejad, M.A., Umstätter, P. and Urbassek, H.M., was originally published Online First without Open Access. After publication in volume 26, issue 3, page 54 the author decided to opt for Open Choice and to make the article an Open Access publication. Therefore, the copyright of the article has been changed to © The Author(s) 2021 and the article is forthwith distributed under the terms of the Creative Commons Attribution 4.0 International License, which permits use, sharing, adaptation, distribution and reproduction in any medium or format, as long as you give appropriate credit to the original author(s) and the source, provide a link to the Creative Commons licence, and indicate if changes were made. The images or other third party material in this article are included in the article’s Creative Commons licence, unless indicated otherwise in a credit line to the material. If material is not included in the article’s Creative Commons licence and your intended use is not permitted by statutory regulation or exceeds the permitted use, you will need to obtain permission directly from the copyright holder. To view a copy of this licence, visit http://creativecommons.org/licenses/by/4.0. Open access funding enabled and organized by Projekt DEAL.

The original article has been corrected.

