# Correction to: Boron nitride nanotubes as containers for targeted drug delivery of doxorubicin

**DOI:** 10.1007/s00894-022-05124-9

**Published:** 2022-06-11

**Authors:** Marjan A. Nejad, Philipp Umstätter, Herbert M. Urbassek

**Affiliations:** Fachbereich Physik Und Forschungszentrum OPTIMAS, Universität Kaiserslautern, Erwin Schrödinger-Straße, 67663 Kaiserslautern, Germany

**Correction to: Journal of Molecular Modeling (2020) 26: 54**

10.1007/s00894-020-4305-z

The article “Boron nitride nanotubes as containers for targeted drug delivery of doxorubicin,” written by Nejad, M.A., Umstätter, P., and Urbassek, H.M., was originally published online first without Open Access. After publication, in volume 26, issue 3, page 54, the author decided to opt for Open Choice and to make the article an Open Access publication. Therefore, the copyright of the article has been changed to © The Author(s) 2021 and the article is forthwith distributed under the terms of the Creative Commons Attribution 4.0 International License, which permits use, sharing, adaptation, distribution, and reproduction in any medium or format, as long as you give appropriate credit to the original author(s) and the source, provide a link to the Creative Commons license, and indicate if changes were made. The images or other third party material in this article are included in the article’s Creative Commons license, unless indicated otherwise in a credit line to the material. If material is not included in the article’s Creative Commons license and your intended use is not permitted by statutory regulation or exceeds the permitted use, you will need to obtain permission directly from the copyright holder. To view a copy of this license, visit http://creativecommons.org/licenses/by/4.0. Open access funding enabled and organized by Projekt DEAL.

In [1], we investigated the diffusion of doxorubicin in water-filled boron nitride nanotubes. Erroneously, Figs. 3 and 4 reported the diffusion in *radial* direction. The figures reproduced in this Erratum display the diffusion in *axial* direction. The numerical values are *D* = 245*.*1 ± 1*.*6 (295*.*6 ± 0*.*7, 357*.*1 ± 1*.*6) μm^2^/s for nanotube radii of *R* = 9*.*0 (12.4, 15.2) Å.

**Fig. 3:**
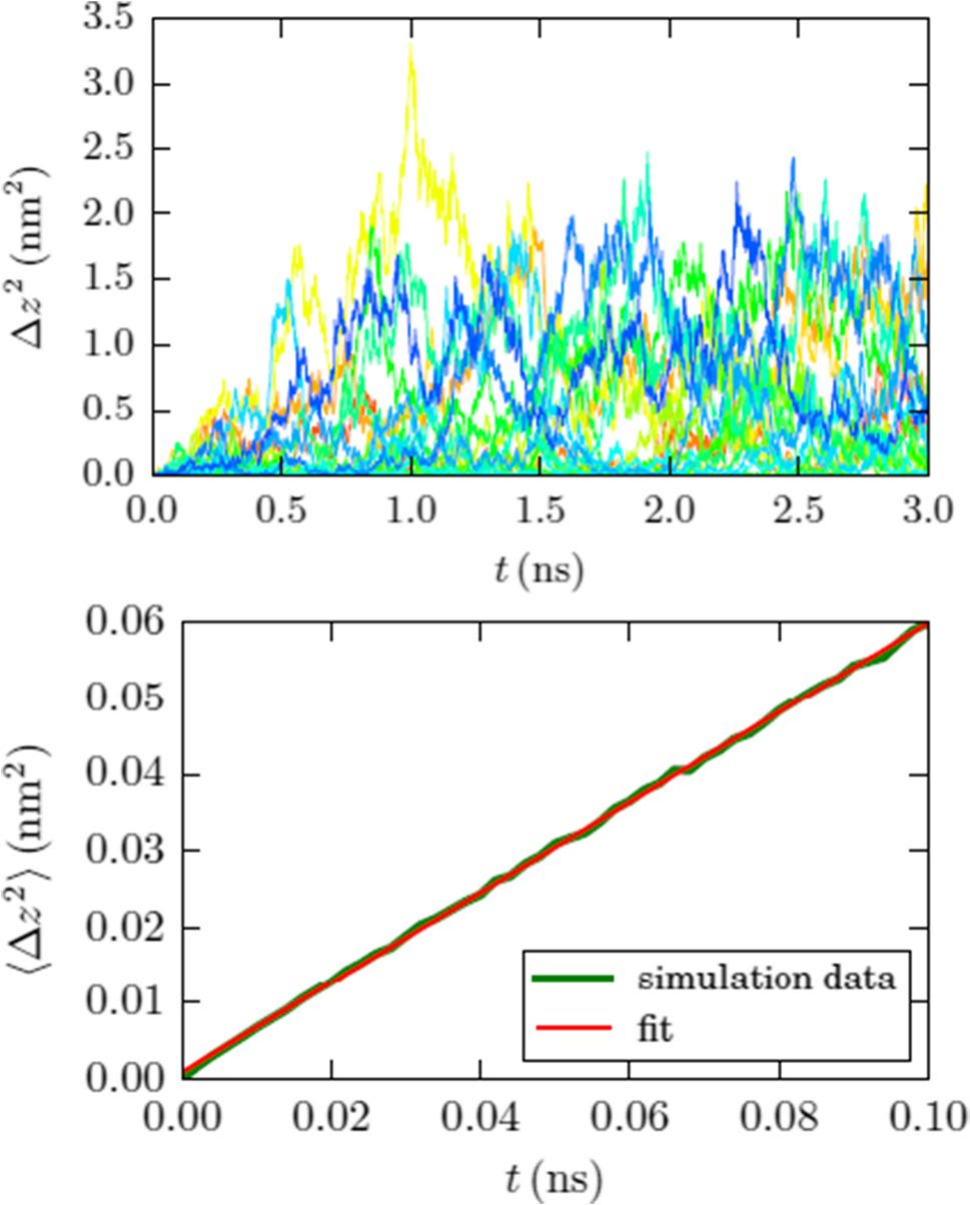
(Revised) Diffusion of doxorubicin in a boron nitride nanotube of radius 12.4 Å. **a** Mean-square displacement of 20 individual 3–ns diffusion runs. **b** Average over the individual runs, compared to a linear fit line

**Fig. 4:**
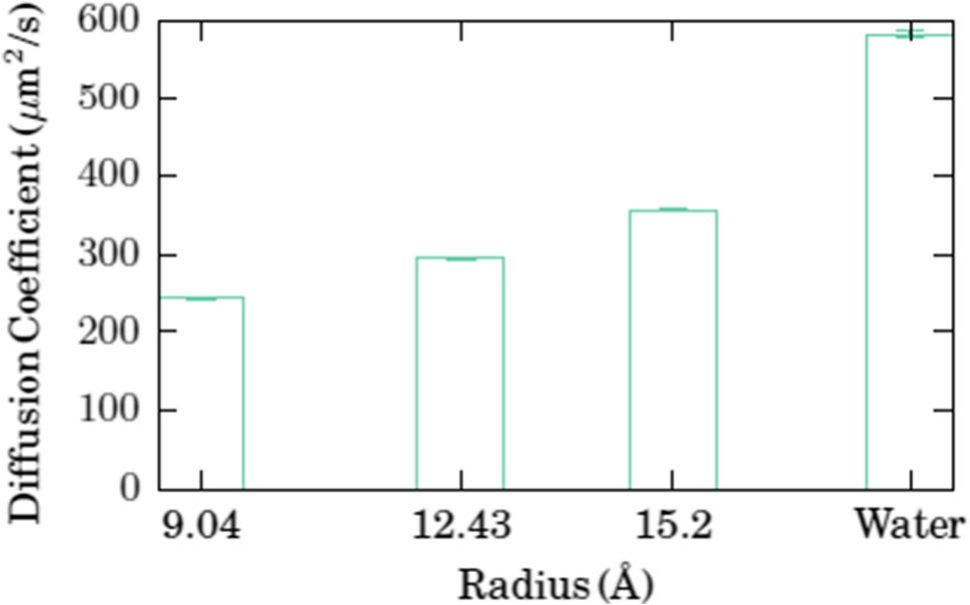
(Revised) Diffusion coefficients of doxorubicin in different nanotubes and in water

The conclusions are unchanged. However, the sentence “Correspondingly, for our narrowest nanotube, we saw almost no diffusion, *D* = 30 μm^2^/s.” should be deleted, and the estimate of the nanotube diameter for which the diffusion coefficient will approach values close to that in pure water is changed from 4 to 2.8 nm, based on a linear extrapolation of the data provided in the revised Fig. 4.

The original article has been corrected.
